# Nano-seq analysis reveals different functional tendency between exosomes and microvesicles derived from hUMSC

**DOI:** 10.1186/s13287-023-03491-5

**Published:** 2023-09-25

**Authors:** Dong Yu, Yue Mei, Ling Wang, Yunpeng Zhao, Xingfei Fan, Dong Liang, Li Li, Jie Zhu, Sisi Bi, Xue Wang, Zhongquan Qi, Lie Zhu, Yue Wang

**Affiliations:** 1 Department of Precision Medicine, Translational Medicine Research Center, Naval Medical University, Shanghai, People’s Republic of China; 2 Department of Stem Cell and Regeneration Medicine, Translational Medicine Research Center, Naval Medical University, Xiangyin Road 800, Shanghai, People’s Republic of China; 3 Department of Histology and Embryology, Basic Medicine Collage, Naval Medical University, Shanghai, People’s Republic of China; 4 Department of Plastic and Reconstructive Surgery, Secondary Affiliated Hospital of Naval Medical University, Shanghai, People’s Republic of China; 5 Medical College of Guangxi University, Nanning, People’s Republic of China; 6 Shanghai Key Laboratory of Cell Engineering, Shanghai, People’s Republic of China; 7 Shanghai Institute of Stem Cell Research and Clinical Translation, Shanghai, People’s Republic of China

**Keywords:** Extracellular vesicles subtypes, Nanopore sequencing, Transcriptomic landscape

## Abstract

**Background:**

Extracellular vesicles (EVs) from human umbilical cord mesenchymal stem cells (hUMSCs) are widely considered to be the best mediators for cell-free therapy. An understanding of their composition, especially RNA, is particularly important for the safe and precise application of EVs. Up to date, the knowledge of their RNA components is limited to NGS sequencing and cannot provide a comprehensive transcriptomic landscape, especially the long and full-length transcripts. Our study first focused on the transcriptomic profile of hUMSC-EVs based on nanopore sequencing.

**Methods:**

In this study, different EV subtypes (exosomes and microvesicles) derived from hUMSCs were isolated and identified by density gradient centrifugation. Subsequently, the realistic long transcriptomic profile in different subtypes of hUMSC-EVs was systematically compared by nanopore sequencing and bioinformatic analysis.

**Results:**

Abundant transcript variants were identified in EVs by nanopore sequencing, 69.34% of which transcripts were fragmented. A series of full-length and long transcripts was also observed and showed a significantly higher proportion of intact or near-complete transcripts in exosomes than that in microvesicles derived from hUMSCs. Although the composition of RNA biotypes transported by different EV subtypes was similar, the distribution of transcripts and genes revealed the inter-heterogeneity and intra-stability between exosomes and microvesicles. Further, 85 different expressed transcripts (56 genes) and 7 fusion genes were identified. Pathway enrichment analysis showed that upregulated-expressed genes in microvesicles were mainly enriched in multiple neurodegenerative diseases, while upregulated-expressed genes in exosomes were mainly enriched in neutrophil extracellular trap formation, suggesting different functional tendencies of EV subtypes.

**Conclusions:**

This study provides a novel understanding of different types of hUMSC-EVs, which not only suggests different transcriptome sorting mechanisms between exosomes and microvesicles, but also shows that different EV subtypes from the same source have different physiological functions, suggesting distinct clinical application prospects.

**Supplementary Information:**

The online version contains supplementary material available at 10.1186/s13287-023-03491-5.

## Introduction

Extracellular vesicles (EVs), act as the major paracrine factor and important mediator for intercellular communication, play a significant role in transferring various bioactive molecules including DNA, RNA, protein, and lipids [1]. Based on the biogenesis mechanism, EVs can be divided into two types, exosome, and ectosome. Based on physical characteristics, EVs are divided into apoptotic vesicles (100–5000 nm), exosomes (50–150 nm), and microvesicles (100–1000 nm) [2]. Among these EVs, exosomes and microvesicles have gained substantial attention due to their therapeutic benefits in various fields.

Umbilical cord mesenchymal stem/stromal cells (UMSCs) are considered to be the most potentially valuable cells for clinical applications due to their remarkable capabilities of self-renew, differentiation, proliferation, and properties that can be easily obtained by non-invasive sampling and in vitro expansion. The potential role of EVs derived from hUMSCs (hUMSC-EVs) has garnered increasing attention in recent years, since its great prospects for the cell-free bio-therapeutic treatment and mitigation of multiple diseases, including various brain pathologies[3], bone repair [4], physical injuries [5, 6], optic nerve injury [7], HCV infection [8].

Multiple studies have highlighted the presence of diverse types of RNAs with UMSC-EVs. The transfer of these RNAs to target cells enables the regulation of gene expression and cellular function, thereby altering the behavior of target cells [9]. UMSC-derived exosomes were found to release lncRNA MALAT1, leading to inhibit the NF-κB/TNF-α signaling pathway, thereby prevent aging-induced cardiac insufficiency [10]. Additionally, miR-21 and miR-23, both highly enriched in UMSC-EVs, were shown to alleviate pulmonary fibrosis by inhibiting the transforming growth factor-β (TGF-β) signaling pathway [11]. Increased levels of serum exosomal PLA2G10 mRNA and protein levels have been confirmed to be associated with more aggressive features of non-small cell lung cancer [12].

Although various NGS-based studies on the transcriptomic profile of EVs have been conducted, most have focused on small RNAs, such as miRNA [13] and circular RNA [5]. It is well known that long-read transcripts play an important role in expression regulation, which may be a vital support for the biological function of EVs. Unfortunately, few studies have focused on long transcripts of EVs. Notably, due to the short-read sequencing and alignment strategy of NGS, it is insufficient to accurately and comprehensively reveal the long transcript profile. In addition, the structure integrity and function of RNA in EVs are also poorly understood. Considering the advantages of single molecules and long-read length, third-generation sequencing has been widely applied in other fields. Thus, we recommend its application in the recognition of EVs transcriptome, especially long transcripts.

Therefore, in this study, we performed nanopore sequencing to comprehensively explore the transcript landscape of hUMSC-EVs. Our results demonstrated that nanopore sequencing showed its superiority in identifying long transcripts in EVs compared with NGS. Transcripts in EVs were found to be mainly fragmented and exosomes contain a more significant proportion of full-length transcripts than microvesicles. We also found that the transcripts in exosomes and microvesicles have high inter-group heterogeneity, suggesting the different transcriptome sorting mechanisms. Meanwhile, the transcription profile of exosomes was more consistent and reproducible. Further, the significantly highly expressed genes in microvesicles were mainly enriched in pathways of neurodegenerative diseases, while those in exosomes were enriched in neutrophil extracellular trap formation. Overall, these findings provide insights into the mechanisms underlying the function of EV subtypes and suggest their potential clinical applications.

## Materials and methods

### Cell culture

The human umbilical cord-derived mesenchymal stem cells (hUMSCs) were purchased from Shanghai East Hospital (Shanghai, China). hUMSCs were cultured at 37 °C and 5% CO_2_ in low-sugar DMEM (11,885,084, Gibco) containing 10% fetal bovine serum (FBS, 10,099,141, Gibco).

### Supernatant collection

After hUMSCs were cultured to 70–80% confluence, cells were washed three times with PBS (70,011,044, Gibco,) and re-cultured in the low-sugar DMEM (11,885,084, Gibco) supplemented with exosome-free serum. The supernatant was collected after 48 h of incubation. Exosome-free serum was prepared by ultracentrifugation at 160000 g for 6 h and filtered through a 0.22 μm filter before being added into the medium.

### Isolation of extracellular vesicles

Briefly, supernatants were centrifuged at 300 g for 10 min and at 2000 g for 10 min to remove cells and debris. The supernatant was centrifuged at 15000 g for 40 min and at 120000 g for 1 h to collect large and small extracellular vesicles separately. The resulting microvesicles pellet and exosomes pellet were resuspended in a large volume of PBS followed by ultracentrifugation at 15,000 g (for 40 min) and 120000 g (for 1 h) to wash the sample. All the centrifugations were performed at 4 °C.

### Particle measurement by nanoparticle tracking analysis (NTA)

The number and size of particles were measured by ZetaView® PMX110 (Particle Metrix, Meerbusch, Germany) according to the user manual. Characterization of large extracellular vesicles and small extracellular vesicles was performed by Echo Biotech (Beijing, China) Corp and OBiO Technology (Shanghai, China) Corp separately.

### Single vesicle analysis by transmission electron microscopy (TEM)

Extracellular vesicles were dissolved in PBS and were dropped onto the carbon film copper grid for 1 min. The grids were negatively stained with 10 μL of 2% uranyl acetate. Finally, the grids were examined with a transmission electron microscope. Characterization of large extracellular vesicles and small extracellular vesicles was performed by Echo Biotech (Beijing, China) Corp and OBiO Technology (Shanghai) Corp separately.

### Western blot analysis

Extracellular vesicles were lysed with RIPA buffer supplemented with protease and phosphatase inhibitors. Proteins were separated by polyacrylamide gel and transferred to PVDF membranes (ISEQ00010; Millipore, Germany). The membranes were blocked with 5% BSA in TBST for 1 h and then incubated with primary antibodies at 4 °C overnight. The primary antibodies were anti-CD63 (1:1000 dilution, ab134045, Abcam, UK), anti-TSG101 (1:1000 dilution, ab133586, Abcam, UK), and anti-ALIX (1:1000 dilution, ab275377, Abcam, UK). The membranes were washed three times with TBST before incubation with the secondary antibody for 1 h at room temperature. The secondary antibody was anti-rabbit IgG (horseradish peroxidase-conjugated, 1:5000 dilution, #7074, CST, USA). After being washed with TBST three times, proteins were visualized using an ECL chemiluminescence staining assay kit. Full blot images were provided in supplementary data (Additional file 1: Fig. S1).

### Library construction and nanopore sequencing

Total RNA was extracted from the six samples by exoRNeasy Serum/Plasma Maxi Kit (Qiagen, Germany) and quantified with Nanodrop ONE (ThermoFisher, USA). For obtaining more types of RNA, we optimized the traditional nanopore sequencing library construction method. We add a step before reverse transcription to tail poly(A) for total RNA by *E.* *coli* Poly(A) polymerase (NEB, USA). The direct cDNA sequencing protocol using the Direct cDNA Sequencing Kit (Oxford Nanopore Technologies Ltd., UK) was followed exactly as described by the manufacturer’s modifications. 100 ng poly(A) RNA was treated by Maxima H Minus Reverse Transcriptase (ThermoFisher, USA) for reverse transcription and strand-switching. Maxima H Minus Reverse Transcriptase can ensure high yields of full-length cDNA products. 1 μl RNase Cocktail Enzyme Mix (AM2286, ThermoFisher, USA) was added to the reverse transcription reaction and incubated for 10 min at 37 °C to degrade RNA. Second-strand synthesis was acted by LongAmp Taq Master Mix (NEB, USA) to produce double-strand cDNA with long-read. cDNA samples were performed end repair and dA-tailing by Ultra II End-prep enzyme mix (NEB, USA) through incubating at 20 °C for 5 min and 65 °C for 5 min using thermal cycler C1000 (Biorad, USA). Native Barcodes and adapters were added by Blunt/TA Ligation Master Mix (NEB, USA) and Quick T4 DNA Ligase (NEB, USA), respectively. AMPure XP beads were used for cDNA purifying during the whole experiment. The cDNA from each sample was quantified with Qubit4 using Qubit™ RNA HS Assay kit (Invitrogen, USA). cDNA from each sample was mixed in equal proportions to generate the final library. 12 μl final library with a total of 430 ng cDNA with barcodes was generated by mixing in equal proportions from each sample. AMPure XP beads were used for cDNA purifying during the whole experiment. The library was sequenced by Biomarker Technologies (Beijing, China), and the base calling was performed by Albacore.

### Nanopore sequencing data analysis

Raw reads were filtered with a minimum average read quality score of < 7 and trimmed by porechop (https://github.com/rrwick/Porechop) to remove adaptors. The quality control was performed by NanoQC (https://pypi.org/project/nanoQC). Nanostat (https://pypi.org/project/NanoStat) was used to calculate read length, reads count, and mean read quality. The state of reads was compared among six samples by Nanocomp (https://github.com/wdecoster/NanoComp). The reads were then aligned to Homo sapiens GRCh38 by minimap2 (https://github.com/lh3/minimap2). Nanocount (https://pypi.org/project/NanoCount) was applied to annotate reads and calculate the relative abundance of reads.

### NGS sequencing and data analysis

Libraries for NGS and sequenced by Biomarker Technologies (Beijing, China). Total RNA-seq reads were performed quality control by FastQC (https://www.bioinformatics.babraham.ac.uk/projects/fastqc/). Low-quality reads (*q* < 20 and read length < 36 bp) and adaptors were trimmed with trim_galore (https://www.bioinformatics.babraham.ac.uk/projects/trim_galore). Trimmed reads were aligned to reference genome GRCH38 by HISAT2 (https://github.com/DaehwanKimLab/hisat2) and duplicated by PicardTools MarkDuplicates. RNA level of the genes was quantified as Fragments Per Kilobase of transcript per Million mapped reads (FPKM) with StringTie (http://ccb.jhu.edu/software/stringtie). The differentially expressed genes were carried out by DEseq2 (https://bioconductor.org/packages/release/bioc/html/DESeq2.html). KEGG, Gene Ontology (GO) enrichment analysis in ClusterProfiler package (https://bioconductor.org/packages/release/bioc/html/clusterProfiler.html) and GSEA (http://www.webgestalt.org/) were performed.

### Statistical analysis

Statistical analysis was performed by R packages and GraphPad Prism 9 (GraphPad Software, San Diego, CA, USA). A value of *p* < 0.05 was considered statistically significant. Data were expressed as mean ± SEM.

## Results

### The physical characteristics of extracellular vesicles

**Fig. 1:**
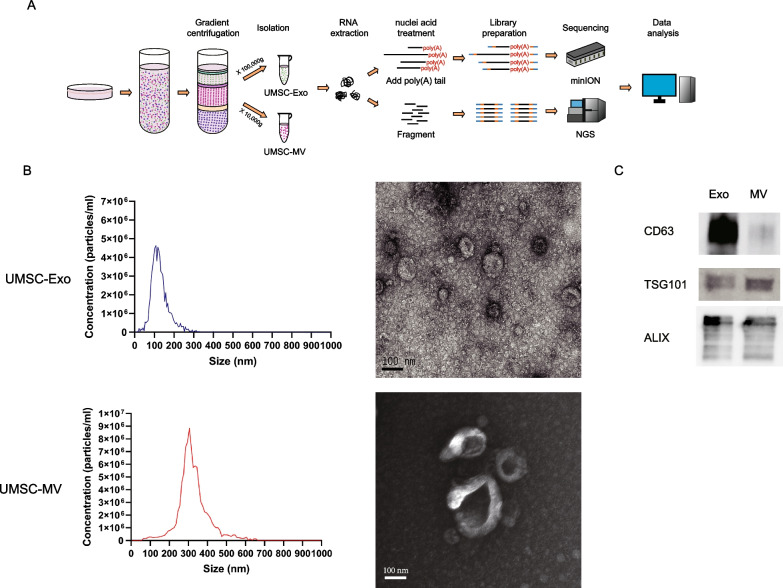
The physical characteristic of extracellular vesicles. **A** Schematic diagram of extracellular vesicles (EVs) transcriptome sequencing workflow, including cell culture, gradient centrifugation, nucleic acid extraction, library construction, sequencing, and data analysis. **B** Physical characterization of umbilical cord-derived mesenchymal stem/stromal cells (UMSCs) derived exosomes (UMSC-Exos) and microvesicles (UMSC-MVs). Size distribution of UMSC-Exos and UMSC-MVs, determined using nanoparticle tracking analyzer (left). Transmission electron microscopy images of UMSC-Exos and UMSC-MVs (right). Scale bar, 100 nm. **C** The identification of biomarkers of EVs determined by Western blot, including CD63, TSG101 and ALIX. Original blot images are presented in Additional file 3: Fig. S3

We proposed an optimized strategy for long-length RNA analysis of extracellular vesicle subtypes derived from hUMSCs (Fig. 1A). The qualitative assessment of EV morphology using transmission electron microscopy revealed that the isolated vesicles were membrane-enclosed. A population of small extracellular vesicles resembling exosomes and a population of larger extracellular vesicles resembling microvesicles were found (Fig. 1B). It was found that exosomes had a narrow particle size distribution with a peak diameter of 127.8 ± 44.6 nm, while microvesicles had a wider particle size distribution with a peak diameter of 324.4 ± 80.4 nm (Fig. 1B) based on Nanoparticle Tracking Analysis, which can calculate the particle size by the rate of particle motion. Meanwhile, Western blot results showed that CD63 was specifically enriched in exosomes, while ALIX and TSG101 displayed no significant difference between exosomes and microvesicles (Fig. 1C, Additional file 1: Fig. S1). This is consistent with the latest research findings that CD63 is the exosome-specific marker, and ALIX and TSG101 are universal markers among extracellular vesicles [14].

### The heterogeneity between exosomes and microvesicles

**Fig. 2:**
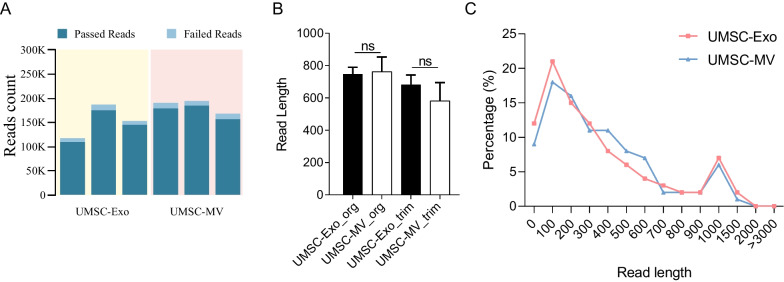
Characteristic of nanopore sequencing data. **A** The reads count of UMSC-Exos and UMSC-MVs based on nanopore sequencing. **B** The read length of UMSC-Exos and UMSC-MVs based on nanopore sequencing. **C** The distribution of read length between UMSC-Exos and UMSC-MVs

To verify the realistic long transcripts in hUMSC-EVs, nanopore sequencing was performed. More than 100,000 reads were obtained for each sample, with an average read quality of more than 11 (Fig. 2A). There was no significant difference in mean (Fig. 2B) and all (Fig. 2C, two-way ANOVA, *p* = 0.888, for microvesicles, *n* = 3, for exosomes, *n* = 3) read length between exosomes and microvesicles. Of exosome reads, 33.339% were less than 200 bp, and 10.05% were longer than 1000 bp. Of microvesicle reads, 28.692% were less than 200 bp, and 8.457% of reads were longer than 1000 bp. The longest read length identified was 31,770 bp in the microvesicles (Table 1).

**Table 1:** Overall characteristics of nanopore sequencing data

Sample	UMSC-Exo			UMSC-MV		
Mean read length	458.4	461.9	381.6	537.2	457.5	300.8
Mean read quality	11.6	11.8	11.8	11.9	12.2	11.8
Median read quality	11.7	11.8	11.8	12	12.2	11.7
Number of reads	92,762	159,617	125,784	149,720	151,759	119,568
Read length N50	791	672	586	784	567	395
the longest read	5366	4382	6911	31,770	4326	3926

*UMSC* Umbilical cord-derived mesenchymal stem/stromal cells, *Exo* Exosome, *MV* Microvesicle

**Fig. 3:**
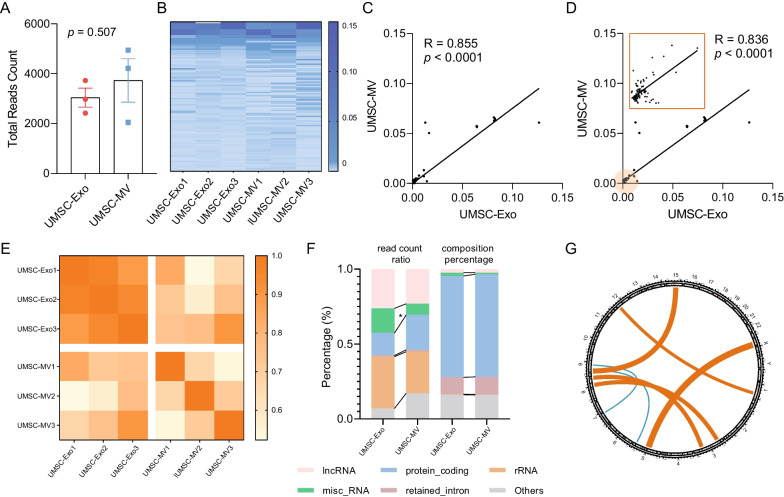
Characteristic of total transcripts in EVs identified by nanopore sequencing data. **A** Total reads count between UMSC-Exos and UMSC-MVs. **B** Heatmap of top 100 transcripts expression. **C** Top 100 transcripts expression correlation diagram. **D** Total transcripts expression correlation diagram. **E** Heatmap of Pearson correlation coefficients of transcripts expression between UMSC-Exos and UMSC-MVs. **F** The distribution of biotypes based on read count ratio and composition percentage. **G** The circular diagram shows the fusion gene between UMSC-Exos and UMSC-MVs

A total of 11,454 transcripts (4077 genes) were identified, and no significant differences in transcript count between exosomes and microvesicles (Fig. 3A, unpaired t test, *p* = 0.507). Among them, only 7.28% of the genes identified in all samples were considered to be intrinsic to EVs (Additional file 2: F Fig. S2A). The chi-square test identified a higher proportion of defined intrinsic genes in exosomes (*p* < 0.0001, chi-square test). Meanwhile, specific transcripts among EV subtypes were identified. 4204 transcripts (1422 genes) were found only in microvesicles, enriching in platinum drug resistance, biosynthesis of cofactors, cellular senescence, and longevity regulating pathways. 3384 transcripts (1113 genes) were found only in exosomes, enriching in ribosome (Table 2).

**Table 2:** Representative enriched pathways of unique genes in exosomes and microvesicles

	ID	Description
UMSC-Exos unique	hsa03040	Spliceosome
UMSC-MVs unique	hsa01524	Platinum drug resistance
	hsa01240	Biosynthesis of cofactors
	hsa04213	Longevity regulating pathway
	hsa04218	Cellular senescence
	hsa03040	Spliceosome
	hsa05213	Endometrial cancer
	hsa05132	Salmonella infection
	hsa04066	HIF-1 signaling pathway

The expression level of total transcripts and top 100 transcripts (Fig. 3B) between microvesicles and exosomes showed a significant correlation (total: *r* = 0.855, *p* < 0.0001, top 100: *r* = 0.836, *p* < 0.0001) (Fig. 3C, D). However, compared with microvesicles, exosomes displayed a higher concordance (Fig. 3E), implying that exosomes had better reproducibility.

Gene annotation revealed that both groups contained abundant types of transcripts, including protein coding, retained intron, lncRNA, nonsense mediated decay, processed transcript, misc_RNA, and rRNA. The composition percentage of transcript types was dominated by protein coding (exceeded 65%), while the proportion of lncRNA, misc_RNA, and rRNA is less than 5%. In terms of reads count ratio, lncRNA, misc_RNA and rRNA accounted for more than 60%, while protein coding accounted for less than 30% (Fig. 3F).

Meanwhile, we identified 7 fusion genes (Fig. 3G, Table 3), of which were shared by the two groups including RAB13-RAB5B, RPL8-PCOLCE2, and 2 were unique to microvesicles.

**Table 3:** Fusion gene detected in extracellular vesicles through nanopore sequencing

Chr	GENE	ALT	GENE	Distribution
1	RAB13	]12:55981068]N	RAB5B	Exo & MV
3		]8:98041854]N	RPL30	Exo & MV
3	PCOLCE2	N[8:144791275[	RPL8	Exo & MV
5	RPS23	]X:70962962]N		Exo & MV
5		N[9:35685270[	TPM2	MV
7	HSPB1	N[9:72008138[		MV
15	ANXA2	N[9:33625355[		Exo & MV

Above all, nanopore sequencing revealed actual and abundant long transcripts in EV subtypes. Various RNA biotypes and fusion genes have been identified. The intra-group consistency and inter-group heterogeneity in different EV subtypes might suggest different sorting mechanisms during the formation of EV subtypes.

### Differences in transcripts between exosomes and microvesicles

We performed differential analysis between exosomes and microvesicles to verify the hypothesis that the transcriptome sorting mechanism is different due to the different formation and physical properties of EV subtypes.

**Fig. 4:**
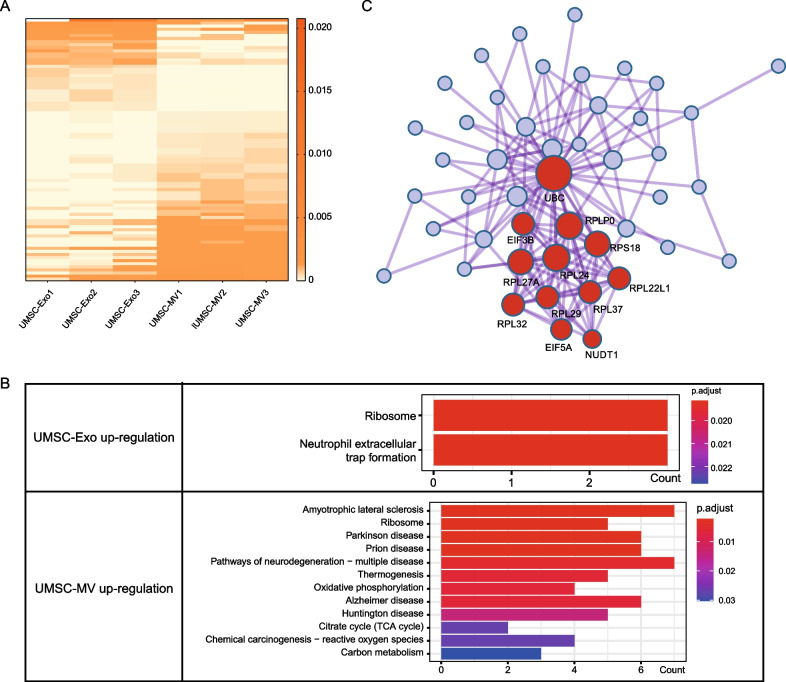
Feature of different expressed transcripts between exosomes and microvesicles. **A** Heatmap of different expressed transcripts between UMSC-Exos and UMSC-MVs. **B** Diagram of pathways differential expressed gene. **C** The gene list with densely connection by the Molecular Complex Detection (MCODE) algorithm

A total of 85 different expressed transcripts (DETs) (56 genes) were identified (Fig. 4A), of which 68.24% were protein coding. Cluster results based on these DETs also showed a higher consistency in each intra-group and a weaker correlation between groups (Additional file 2: Fig. S2B). Among 85 DETs, 30 (19 genes) were upregulated and 55 (38 genes) were down-regulated in exosomes (Table 4). KEGG pathway enrichment analysis showed that 38 down-regulated genes were enriched in multiple neurodegeneration diseases, oxidative phosphorylation, and citrate cycle (TCA cycle), while 19 upregulated genes were mainly enriched in ribosome and neutrophil extracellular trap formation (Table 5, Fig. 4B). For GO pathway analysis, upregulated genes in exosomes were specifically enriched in DNA packaging complex and nucleosome related pathways, while down-regulated were specifically enriched in ATP metabolic process, cellular respiration, unfolded protein binding (Additional file 2: Fig. S2C). Pathway analysis suggests the functional specificity among different EV subtypes.

**Table 4:** Representative different expression genes between exosomes and microvesicles

	RNA types	Representative genes
UMSC-Exos upregulated transcripts (*n* = 30)	Protein coding	EIF5A, CHMP2A and RPL22L1
	Retained intron	HMGN1 and UCHL1
	lncRNA	RPPH1 and CTD-2651B20.6
UMSC-MVs upregulated transcripts (*n* = 55)	Protein coding	CHD4, MTLN, TRIM28, and ZYX
	Retained intron	PSMA4, ACTB and FSCN1
	lncRNA	SNHG32, SNHG29

**Table 5:** Representative enriched pathways of different expression genes

	ID	Description
UMSC-Exos upregulated pathways	hsa03010	Ribosome
	hsa04613	Neutrophil extracellular trap formation
UMSC-MVs upregulated pathways	hsa05014	Amyotrophic lateral sclerosis
	hsa03010	Ribosome
	hsa05012	Parkinson disease
	hsa05020	Prion disease
	hsa05022	Pathways of neurodegeneration—multiple diseases
	hsa04714	Thermogenesis
	hsa00190	Oxidative phosphorylation
	hsa05010	Alzheimer disease
	hsa05016	Huntington disease
	hsa00020	Citrate cycle (TCA cycle)
	hsa05208	Chemical carcinogenesis—reactive oxygen species
	hsa01200	Carbon metabolism

Meanwhile, protein–protein interaction (PPI) analysis showed dense interactions among these genes (Additional file 2: Fig. S2D), in which ACTB, PPIA, RPL32, RPL24, EIF5A, EIF3B, HSPE1, RPLPO acted as the key nodes. The Molecular Complex Detection (MCODE) algorithm was also applied to identify the subnetwork with densely connection (Fig. 4C). The largest subnetwork included a set of ribosomal proteins, EIF5A, EIF3B, NUDT1, and UBC. EIF5A and EIF3B are members of eukaryotic translation initiation factors. EIF3B, down-regulated in exosomes, plays the main role in regulating the interaction between ribosomes and microRNAs during the initial process of protein synthesis. It has been reported that inhibition of EIF3B expression could significantly inhibit proliferation and increase apoptosis of ovarian cancer cells [15].

In addition, 4 significantly differentially expressed lncRNAs were identified, among which CTD-2651B20.6 (unpaired *t* test, *p* = 0.022) and RPPH1 (unpaired *t* test,* p* = 0.034) were highly expressed in exosomes, while SNHG29 (unpaired *t* test, *p* < 0.0001) and SNHG32 (unpaired* t* test, *p* = 0.007) were highly expressed in microvesicles (Additional file 2: Fig. S2E). It is reported that RPPH1 promotes exosome-mediated macrophage M2 polarization [16], while SNHG29 regulates multiple pathways, including YAP [17], Wnt [18], α-Klotho/FGFR1/FGF23 axis [19].

Overall, functional analysis based on differentially expressed transcripts suggests that different EV subtypes may have different functions and potential applications.

### Verification of RNA integrity in extracellular vesicles

The accuracy of the nanopore sequencing data was verified by using NGS data from the same batch of samples and public databases. 89.43% and 77.8% of genes identified by nanopore sequencing were also found in public databases (exoRBase2, Vesiclepedia, and ExoCarta) and NGS (Additional file 2: Fig. S2F), respectively.

**Fig. 5:**
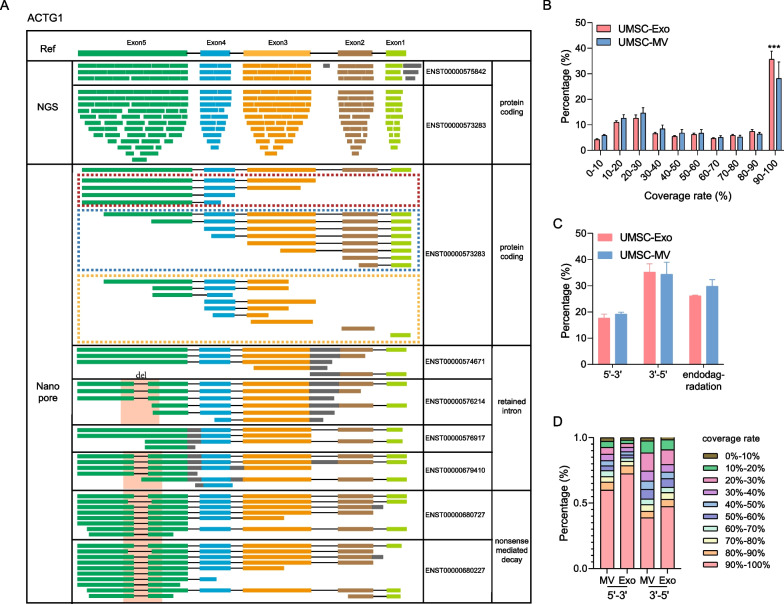
Signatures of fragmented RNAs. **A** ACTG1 transcripts identified by third-generation sequencing and NGS. Blue boxes represent exon5. Blue boxes represent exon4. Orange boxes represent exon3. Brown boxes represent exon2. Green boxes represent exon1. Gray boxes represent retained introns. Black lines skipped introns. Red background frames represent deletion in exon5. Red dashed box represents RNAs under 3′ to 5′ degradation. Blue dashed box represents RNAs under 5′ to 3′ degradation. Orange dashed box represents RNAs under endodegradation. **B** The distribution of RNAs vs. reference transcripts coverage. **C** The distribution of fragmented RNAs direction. **D** The coverage rate distribution of fragmented RNAs under 3′ to 5′ or 5′ to 3′ degradation

Nanopore sequencing identified more abundant transcript variants, of which only 14.96% were also identified in NGS. This may be attributed to the fact that NGS is short-read sequencing, and each read can only reveal sequence information of about 400 bp. Meanwhile, the alignment strategy for NGS is to obtain short-read composition pattern with high probability, which cannot accurately present the sequence of each real transcript. However, nanopore sequencing is direct single-molecule sequencing, which allows the most authentic RNA variants to be identified. Taking ACTG1 as an example, 22 transcript variants were identified by nanopore sequencing, while only two variants were detected by NGS (Fig. 5A). Nanopore sequencing specifically identified the transcript variants with retained introns (ENST00000574671, ENST00000576214, ENST00000576917, ENST00000679410) and partial deletions of exons due to alternative splicing (ENST00000680727 and ENST00000680227). These events can interrupt the main open reading frame of mRNAs, leading to the introduction of premature termination codons, which in turn affect protein translation. In summary, nanopore sequencing showed advantages in transcript identification.

We then investigated the integrity of total transcripts in EVs and found that RNAs in EVs detected by nanopore sequencing were predominantly fragmented (69.34%), and quite a bit of intact transcripts was also observed (30.66%). The distribution of transcript length showed that the proportion of intact or close complete transcripts (coverage rate > 90%) in exosomes is significantly higher than in microvesicles (*t* test, *p* = 0.0002) (Fig. 5B), indicating that functional RNAs carried in exosomes were more abundant. Notably, the genes with full-length transcript in exosomes and microvesicles showed different functional tendencies. Bacterial invasion of epithelial cells and pentose phosphate pathway was found to be uniquely enriched in exosomes, while RNA degradation, nucleocytoplasmic transport, and protein export were unique in microvesicles (Table 6).

**Table 6:** Representative enriched pathways of genes with intact transcripts

	ID	Description
UMSC-Exos unique pathways	hsa05100	Bacterial invasion of epithelial cells
	hsa00030	Pentose phosphate pathway
UMSC-MVs unique pathways	hsa00020	Citrate cycle (TCA cycle)
	hsa03430	Mismatch repair
	hsa00480	Glutathione metabolism
	hsa03420	Nucleotide excision repair
	hsa03013	Nucleocytoplasmic transport
	hsă0	Protein export
	hsa03018	RNA degradation
	hsa01210	2-Oxocarboxylic acid metabolism

These results demonstrated the advantages of third-generation sequencing in identifying RNA integrity and long transcripts compared to NGS, which can more accurately and comprehensively reflect the true structure of transcripts in vesicles.

### The direction of RNA degradation in extracellular vesicles has a bias

Considering the potential role of fragmented transcripts in the transcription and translation phases, we explored the profile of fragmented transcripts and found their breaks were mainly directional, which was consistent with the typical RNA degradation mechanisms. Taking ENST00000573283 as an example, the mapped reads, covering partial exon 3 and completed exon 4–5, or partial exon1 and completed exon 2–5, were likely to be generated by 3′ to 5′ degradation. The reads consisting of complete exon 1–3 or exon 1–2 was the production under RNA 5′ to 3′ degradation. The reads, composed of exon 3–4, might be the RNA endodegradation products (Fig. 5A). Meanwhile, we also observed the expression of enzymes involved in RNA degradation in EVs, including endoribonuclease SMG5, PARN, CNOT1, and the assembly factors LSM family. Further, we found that 3′ to 5′ degradation accounted for the highest (42.66%), followed by endodegradation (34.47%), and 5′ to 3′ degradation was the lowest (22.88%) (Fig. 5C). Notably, the reads with high coverage of corresponding transcripts were mainly present in 3′ to 5′ or 5′ to 3′degradation (Fig. 5D). Meanwhile, the patterns of 3′ to 5′ and 5′ to 3′ degradation varied between exosomes and microvesicles.

## Discussion

According to the European Regulation 1394/2007/EC, human umbilical cord-derived mesenchymal stem/stromal cells (hUMSCs) are classified as advanced therapeutic medicines, due to their unique properties, including self-renewal, pluripotency, and accessibility, as well as their immunosuppressive capacity and lower ethical concerns [20]. A growing number of studies have shown that extracellular vesicles (EVs) have become an important mediator of intercellular communication, not only participating in normal physiological processes but also playing a crucial role in the development and progression of diseases. Compared with whole-cell-based therapies, hUMSC-EVs show great potential, including a lower propensity to trigger innate and adaptive immune responses, the inability to directly form tumors, and safer storage without loss of function [21]. However, there is no clear understanding of the composition of hUMSC-EVs. Our study is the first to comprehensively analyze the transcriptome of different EV subtypes derived from hUMSCs and their biological functions, providing new insights into the heterogeneity of EVs and guidance for clinical applications.

Despite the rapid and multifaceted expansion of EV research in recent years, the vast majority of knowledge about transcripts in EVs focuses on miRNA based on next-generation sequencing (NGS). Third-generation sequencing focus on single molecule and can obtain the complete sequence information, especially for long RNAs. Based on our results, abundant types of long-read RNAs, including protein coding transcripts (mRNAs) and abundant types of noncoding RNAs, were identified in EVs. These RNAs in EVs can be classified into three types: intact RNAs with established functions, such as intact mRNA (e.g., NET1) and lncRNA (e.g., CH507-513H4.4); intact RNAs with unknown functions, such as misc_RNA; and RNA fragments produced by degradation, such as mRNA fragments, some of which might be functional. Several studies have concluded that long RNAs are stably present in EVs and function in recipient cells [22, 23]. The lncRNAs in EVs cannot only act as sponges for microRNAs, whereby microRNAs are loaded into EVs [24] but also function in biological processes, such as the incorporation of hypoxia-inducible factor 1α-stabilizing lncRNAs into EVs released by tumor-associated macrophages to support breast cancer cell viability [4]. Gluc mRNA overexpressed by glioblastoma cells is transported to recipient human brain microvascular endothelial cells by EVs and expresses the protein [25]. Hadi Valadi has confirmed that after transferring mouse exosomal RNA to human mast cells, new mouse proteins were found in the recipient cell [26]. Batagov found 3′-end–derived mRNA fragments in exosomes [27]. The function of various RNAs in hUMSC-EVs in recipient cells requires more experiments to verify.

Transcriptome studies based on NGS have shown that the cellular compositions are not randomly assigned into EVs, but that specific subsets of RNA, proteins, and lipids are incorporated based on an active sorting mechanism [28, 29]. Several studies have found that the miRNA and mRNA expression profiles of exosomes differ from those of the parent cells [25, 30]. Guduric-Fuchs and his team found that a subset of miRNAs (e.g., miR-150, miR-142-3p, and miR-451) preferentially enter EVs [31]. EV subtypes differ in physical properties and biogenesis patterns [21], in which exosomes are endosomal-derived, generated, and released by the fusion of plasma membrane and multivesicular bodies, and microvesicles are produced by outward budding and fission of the plasma membrane. In our result, the profile of RNA in exosomes differs from microvesicles’ RNA content, both in terms of biotypes and specific sequences, which reveals that distinct EV subtypes harbor a specific subset of RNAs rather than random cellular components, suggesting different RNA sorting mechanisms during their formation. Meanwhile, higher intra-group expression consistency was found in exosomes, suggesting that exosomes can be used as a more reproducible and stable cell-free therapeutic medium in clinical applications.

The functional analysis based on the genes in EV subtypes suggests different potential clinical applications. We found a series of full-length transcripts unique to different EV subtypes. VEGFA, only found in MVs, is a high specific growth factor, which can promote target cell survival and proliferation. EIF4G1, EIF2B4, and EIF3D, only found in MVs, are both eukaryotic translation initiation factors, which might regulate gene expression in target cells. SNRPA, SNRNP40, RBMX, DHX38, PPIL1, HNRNPA3, only found in exosomes, are both associated with pre-mRNA splicing. Sergio T. Ferreira’s group has revealed that EVs derived from human Wharton’s jelly MSCs have neuroprotective effects and can prevent neuronal damage in Alzheimer’s disease [32]. It has also been reported that MSCs-derived exosomes can exert neuro-regenerative effects [33]. MSCs-derived exosome treatment has been confirmed to enhance cognitive function and promote adult neural stem cell differentiation in adult mice [34]. In our result, differentially highly expressed genes in exosomes are primarily enriched in neutrophil extracellular trap formation (NET), which is considered to capture and kill bacteria, and impact the tumor microenvironment [35]. Therefore, exosomes derived from hUMSC are believed to be more advantageous in the therapy of tumors and bacterial infections. Meanwhile, the differentially highly expressed genes in microvesicles are mainly enriched in multiple neurodegenerative diseases, prion diseases, and some physiological processes, implying that microvesicles derived from hUMSC have greater potential for neurodegenerative diseases, aging, and cancer therapy. Functional similarity of the genes SDHB [36], BAD (a member of BCL-2 family) [37], and PSMA4 [38], associated with neuroprotection and neurodegeneration, enriched in different EV subtypes suggest different clinical application prospects. Moreover, the wound healing and cell proliferation assay suggest exosomes and microvesicles derived from hUMSCs have different effects on migration and proliferation of HUVEC cell lines (Additional file 3: Fig. S3).

To our knowledge, this is the first study demonstrating the long transcriptomic landscape of hUMSC-EV subtypes based on third-generation sequencing, composed of abundant fragmented transcripts. The transcriptomic profile is inter-heterogeneous between exosomes and microvesicles, suggesting inconsistent RNA sorting mechanisms. Functional enrichment shows various tendencies in clinical applications for exosomes and microvesicles. In addition, the transcriptomes encapsulated in exosomes are more consistent, stable, and reproducible and contain more functional full-length transcripts, suggesting a stronger clinical potential.

## Conclusions

Overall, in the study, we successfully isolated and identified exosomes and microvesicles derived from hUMSCs and systematically compared long transcriptomic profile by nanopore sequencing. Different transcriptome sorting mechanisms and application prospects of EV subtypes were found. It would provide references for the basic research and clinical applications in future.

### Supplementary Information

**Additional file 1**. The identification of exosomes and microvesicles in protein level.

**Additional file 2**. Characteristic of different expressed transcripts between exosomes and microvesicles.

**Additional file 3**. Functional verification of exosomes and microvesicles on HUVEC cell line.

## Data Availability

RNA-seq data based on NGS and third-generation sequencing are publicly available in the CNSA (https://db.cngb.org/cnsa/) of CNGBdb under the accession number CNP0002962.

## Abbreviations

hUMSCs: Human umbilical cord-derived mesenchymal stem/stromal cells
EV: Extracellular vesicle
