# Supplementary material for: Nano-seq analysis reveals different functional tendency between exosomes and microvesicles derived from hUMSC
Source: Stem Cell Res Ther. 2023 Sep 25;14:272. doi: 10.1186/s13287-023-03491-5 (PMC10521478; doi:10.1186/s13287-023-03491-5)
Supplement: Supplementary file 1 — Additional file 1. The identification of exosomes and microvesicles in protein level. [file 13287_2023_3491_MOESM1_ESM.pdf]

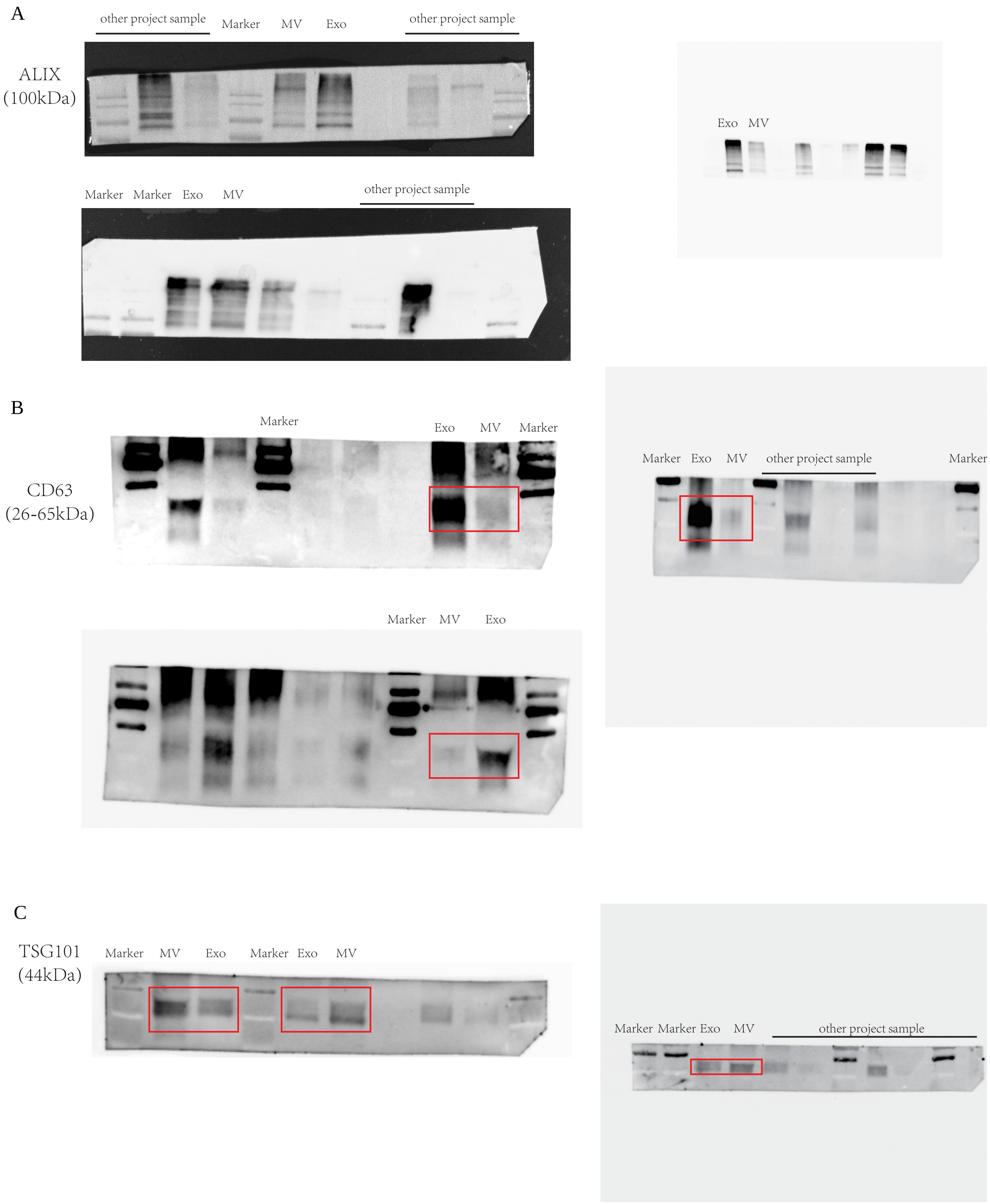

Figure S1 The original western blot of ALIX (A), CD63 (B) and TSG101 (C) protein expression between exosomes and microvesicles derived from hUMSC.
