# Supplementary material for: Nano-seq analysis reveals different functional tendency between exosomes and microvesicles derived from hUMSC
Source: Stem Cell Res Ther. 2023 Sep 25;14:272. doi: 10.1186/s13287-023-03491-5 (PMC10521478; doi:10.1186/s13287-023-03491-5)
Supplement: Supplementary file 2 — Additional file 2. Characteristic of different expressed transcripts between exosomes and microvesicles. [file 13287_2023_3491_MOESM2_ESM.pdf]

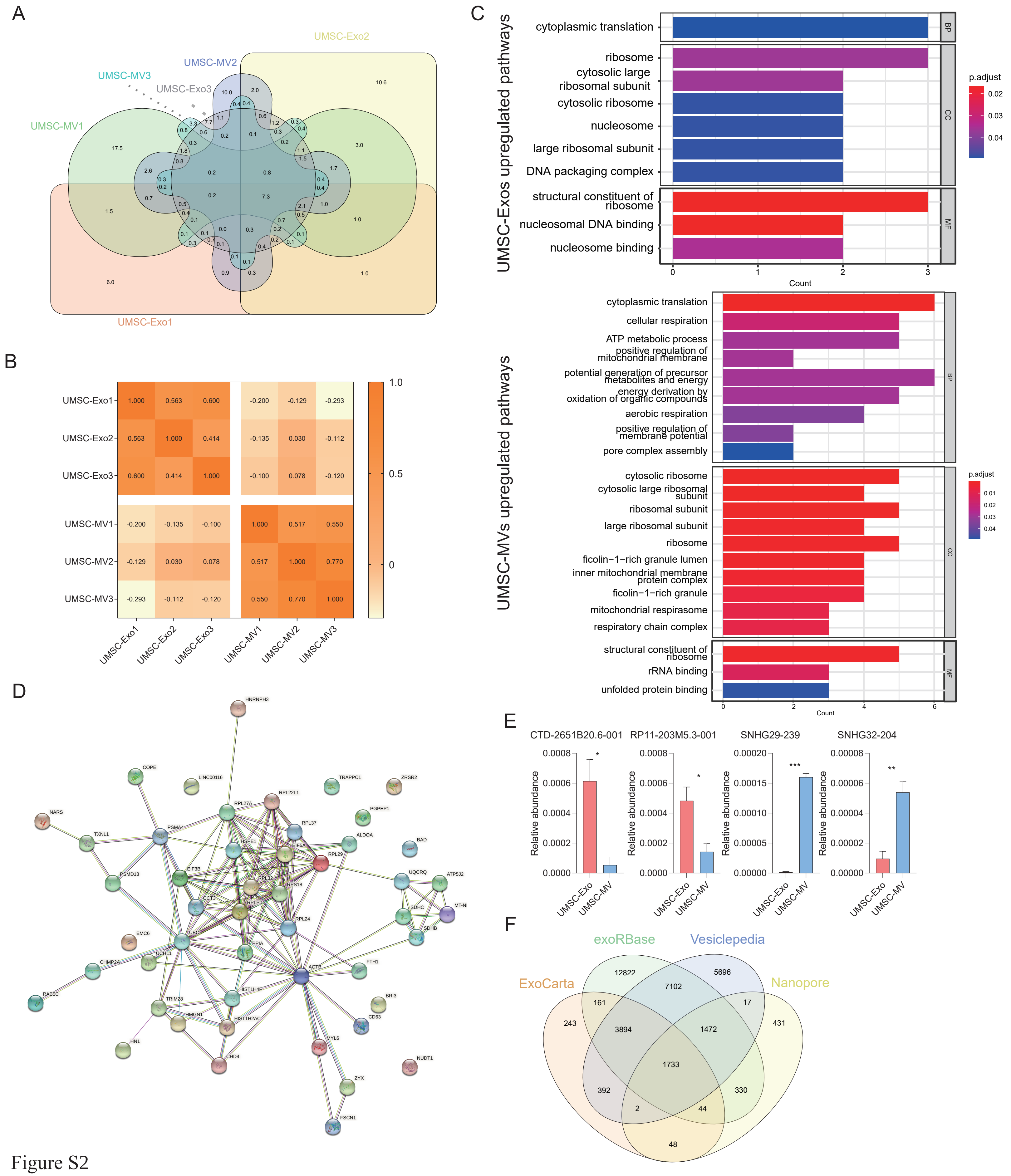

Figure S2

(A) The transcript distribution between exosomes and micro-vesicles. (B) Heatmap of Pearson correlation coefficients of different expressed transcripts between exosomes and micro-vesicles. (C) Diagram of pathways differential expressed gene. (D) Establishment of PPI network of the differential expressed genes using STRING database. (E) The expression of four differentially expressed lncRNAs. (F) The gene distribution between public databases and nanopore sequencing.
