# Supplementary material for: Nano-seq analysis reveals different functional tendency between exosomes and microvesicles derived from hUMSC
Source: Stem Cell Res Ther. 2023 Sep 25;14:272. doi: 10.1186/s13287-023-03491-5 (PMC10521478; doi:10.1186/s13287-023-03491-5)
Supplement: Supplementary file 3 — Additional file 3. Functional verification of exosomes and microvesicles on HUVEC cell line. [file 13287_2023_3491_MOESM3_ESM.pdf]

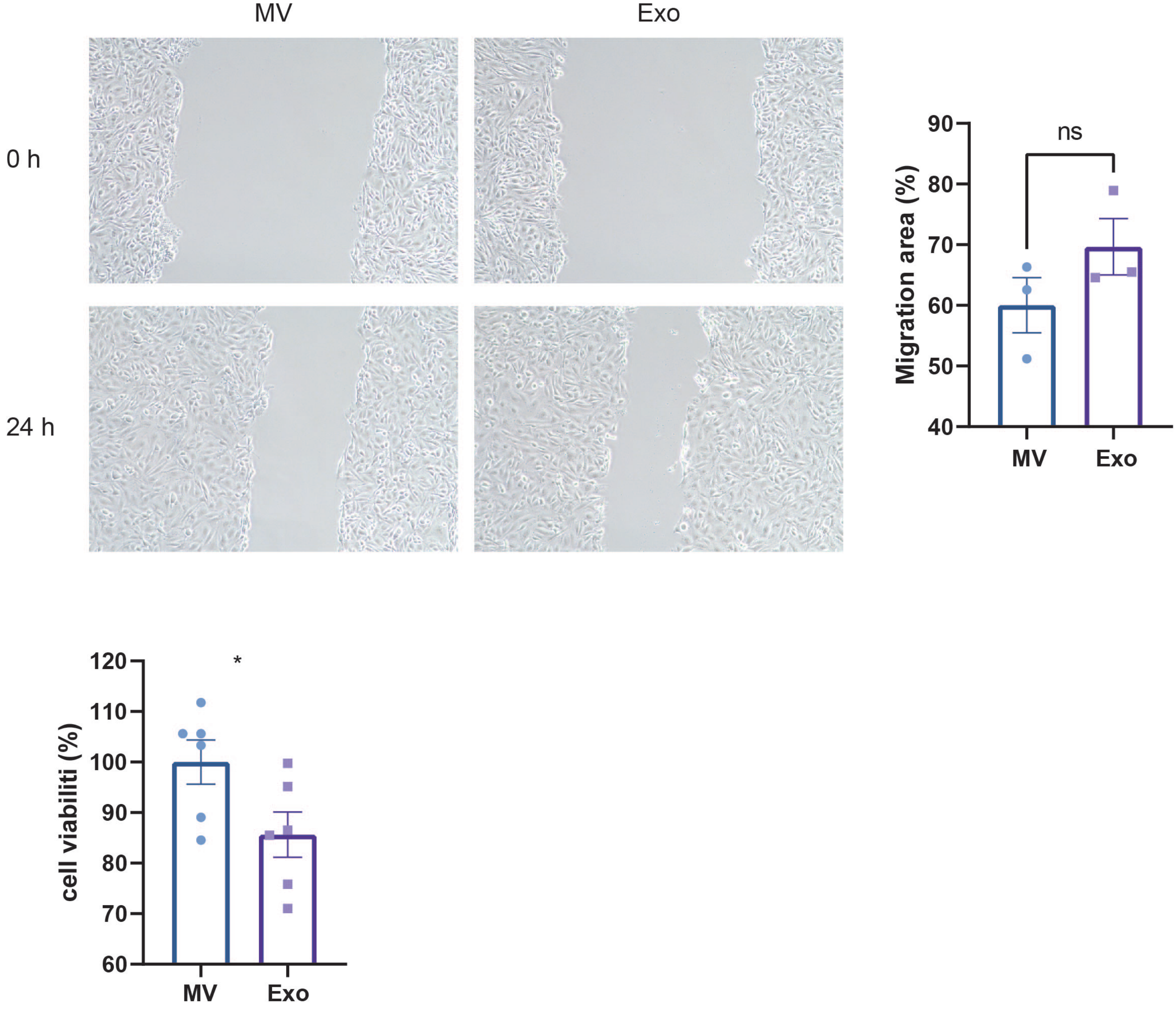

Figure S3
(A) Wound healing assay of HUVEC cell line co-cultured with exosomes and micro-vesicles derived from hUMSC respectively. (B) Cell proliferation assay of HUVEC cell line co-cultured with exosomes and micro-vesicles derived from hUMSC respectively.
